# Intraosseous Ganglion of the Distal Tibia: Clinical, Radiological, and Operative Management

**DOI:** 10.1155/2015/759257

**Published:** 2015-01-13

**Authors:** Sedeek Mohamed Sedeek, Q. Choudry, S. Garg

**Affiliations:** ^1^Department of Orthopaedics, East Lancashire Hospitals NHS Trust, Blackburn BB2 3HH, UK; ^2^Department of Orthopaedics, Royal Lancaster Infirmary, Lancaster LA1 4RP, UK

## Abstract

Intraosseous ganglia are benign cystic lesions located in the subchondral bone. Intraosseous ganglion cysts of the ankle are relatively uncommon. We present a case of recurrent intraosseous ganglion in the ankle of a 41-year-old female who had recurrence after initial surgery. She was treated effectively by curettage and autogenous cancellous bone grafting. At the final follow-up, satisfactory results were obtained with no recurrence or complications.

## 1. Introduction

Ganglia are common and usually occur near joints, tendons, and tendon sheaths. However, the occurrence of intraosseous ganglions is rare [1, 2]. Intraosseous ganglia are benign cystic and often multiloculated lesions composed of fibrous tissue with extensive mucoid changes located in the subchondral bone adjacent to the joints [2, 3].

Intraosseous ganglia occur in the mature skeletons of patients of all ages and have a peak incidence in the fourth and fifth decades of life [4]. The femoral head and tibia are commonly affected. However, the occurrence in the subchondral region of the ankle is rare, with only few reports in the literature [5].

This paper presents a case of recurrent intraosseous ganglion in the ankle of a 41-year-old female who was treated effectively by curettage and autogenous cancellous bone grafting.

## 2. Case Study

**Figure 1:**
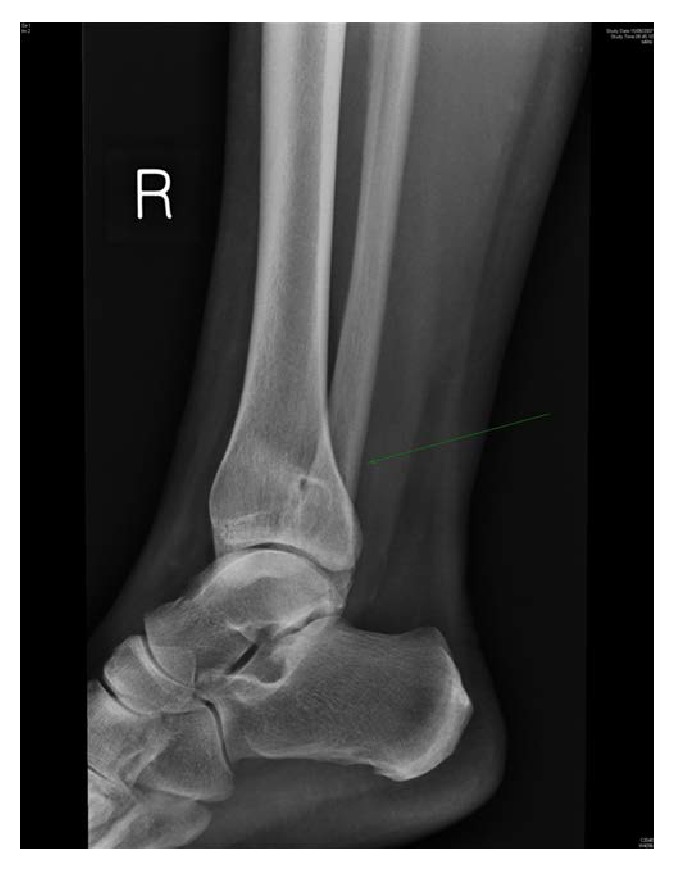
Lateral view X-ray of the ankle showing well defined cystic lesion in distal tibia.

**Figure 2:**
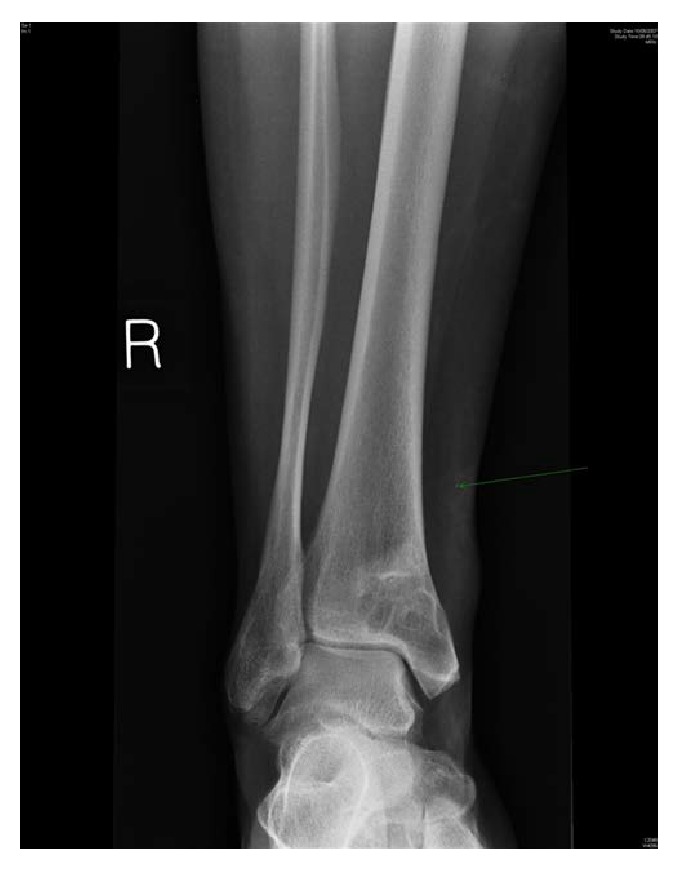
AP X-ray of ankle showing cystic lesion in distal tibia.

A 41-year-old woman presented with a 2-year history of right ankle pain and limping which had deteriorated in the past 6 months. There was no history of trauma. On examination, there was no effusion swelling or tenderness of the right ankle. The patient had full range of motion in her ankle, and there was no vascular or neurological abnormality. Inflammatory blood markers were elevated. Plain radiographs demonstrated a cystic lesion of the lower tibia extending into the medial malleolus (Figures 1 and 2). Benign cyst, nonossifying fibroma, and Brodie abscess were considered in the differential diagnosis. A Brodie abscess was thought to be the likely cause and she underwent percutaneous drilling under image guidance. Intraoperatively there was no pus detected; a swab for culture and sensitivity was taken. No organisms were grown from the microbiology swab specimen. Postoperatively she was afebrile and able to mobilise and was discharged home with a simple dressing. At follow-up, the patient was asymptomatic. The cavity was radiologically filled and she returned to work.

**Figure 3:**
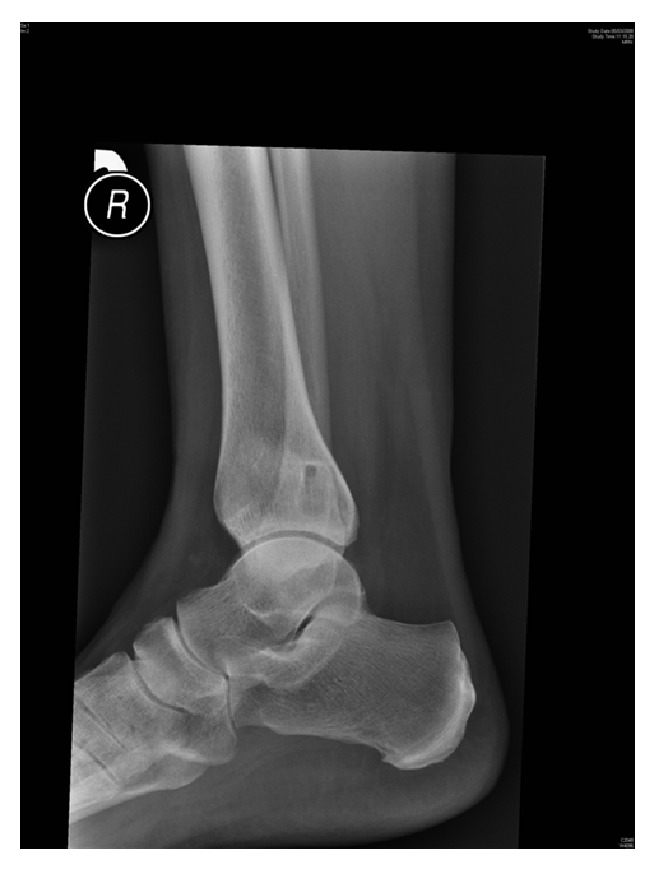
Lateral view X-ray showing reoccurring cystic lesion in distal tibia.

**Figure 4:**
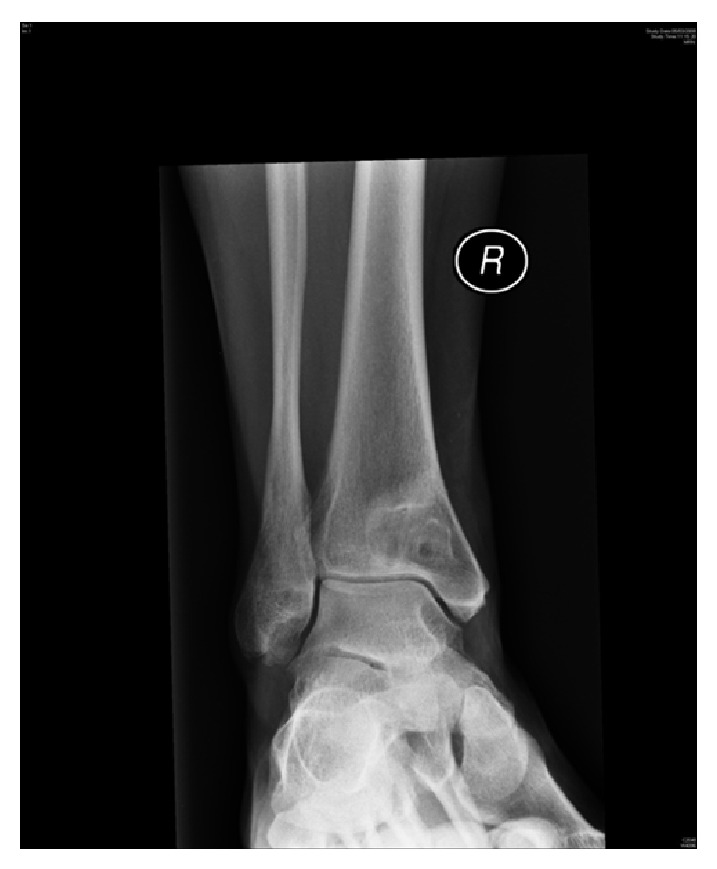
AP X-ray showing reoccurrence of cystic lesion distal tibia.

**Figure 5:**
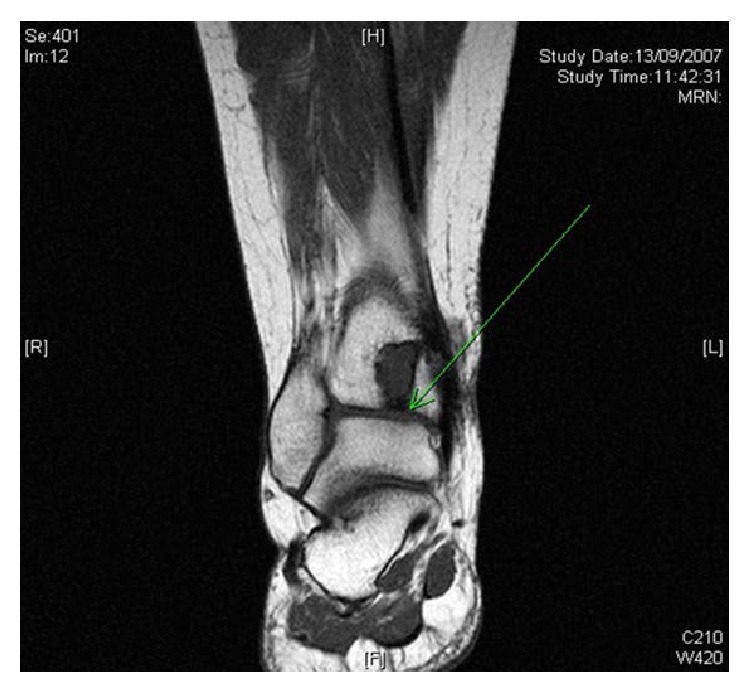
T1 weighted MRI showing 1.5 cm intracortical, subarticular cyst.

**Figure 6:**
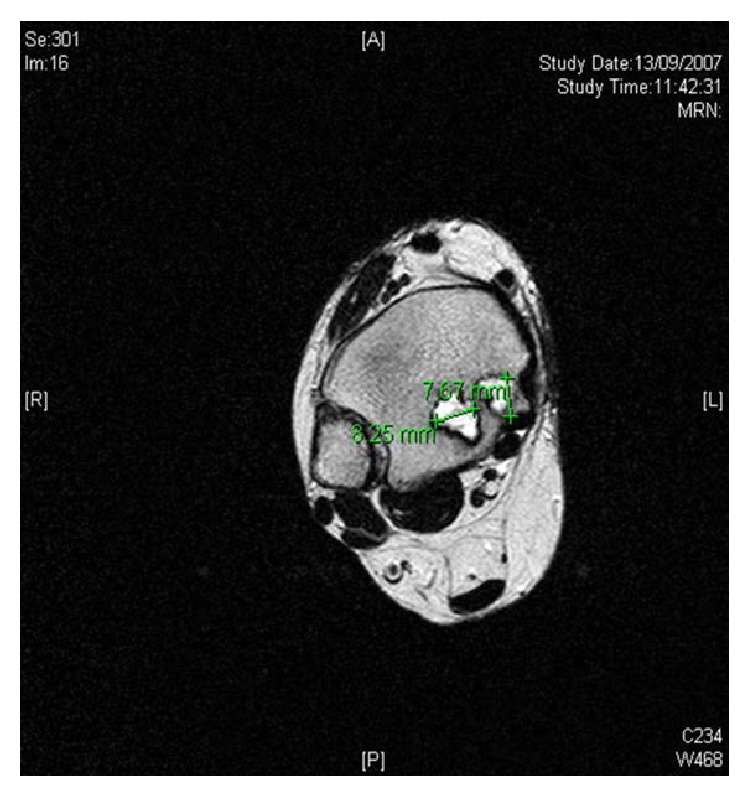
Axial view MRI showing well defined cystic fluid filled lesions in distal tibia.

Four years later, the patient was referred by a general practitioner for the recurrence of ankle pain. The examination revealed mild tenderness over the lower tibia and above the medial malleolus; however, there was no soft-tissue swelling. Her X-rays revealed a recurrence of the osteolytic lesion in the distal tibia (Figures 3 and 4). Magnetic Resonance Imaging (MRI) demonstrated a well-circumscribed cystic-like lesion in the lower tibia, just above the joint surface (Figures 5 and 6). Surgery was planned with anteromedial approach to the right ankle performed under general anaesthesia and X-ray guidance. The periosteum was reflected from the medial malleolus, and a trap door window was cut over the lesion. A cyst with a fibrous wall was found measuring approximately 2 × 1 cm. The cyst contained clear fluid. The cavity was currettaged and the roof was removed. The cavity was then packed with iliac crest bone graft. There was no direct communication between the cavity and the ankle joint. Histological examination showed fibrous tissue with myxoid changes consistent with an intraosseous ganglion.

**Figure 7:**
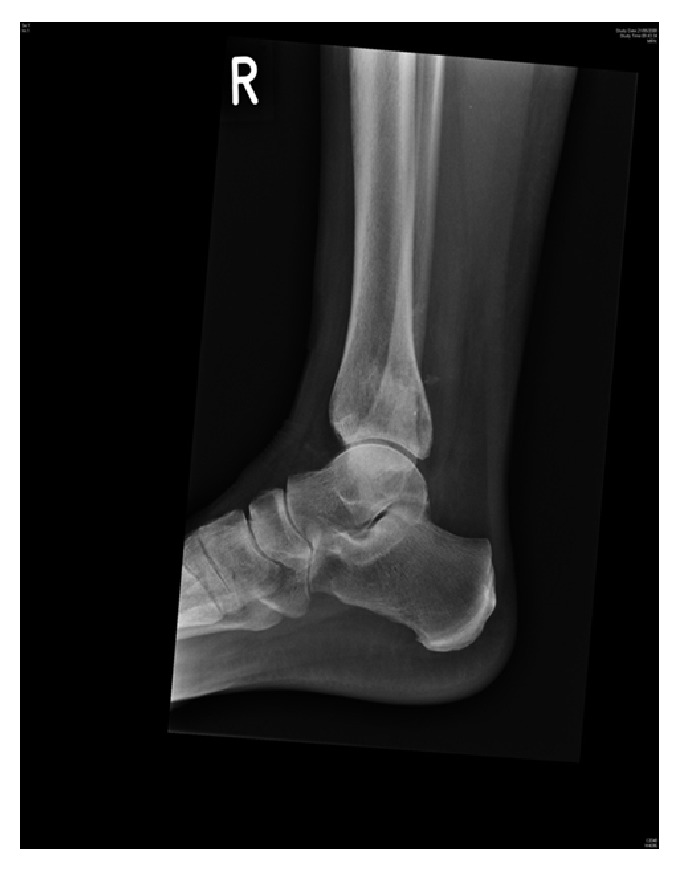
Lateral view X-ray at follow-up showing complete incorporation of graft and healing of lesion.

Postoperatively, a non-weight-bearing short-leg cast was applied to protect the ankle joint. The cast was removed at 6 weeks and physiotherapy commenced. Three months later the patient returned to full work. At 4 months after surgery her X-rays demonstrated complete graft incorporation and healing (Figure 7).

At the latest follow-up (2 years postoperatively), the patient had no pain or limitation of movement and there was no further local recurrence.

## 3. Discussion

An intraosseous ganglion cyst is an uncommon, benign cystic lesion of the bone occurring in close relationship to an articular surface [5]. The condition has a minor male preponderance. The youngest reported patient in the literature was aged 18, while the oldest patient was 86. Most patients are in the middle-age group [3, 6, 7]. The aetiology and pathogenesis of intraosseous ganglions remain unknown. There have been several theories about the pathogenesis of the cyst [4, 8, 9]. Schajowicz et al. suggested 2 types of intraosseous ganglion. The first is a primary or idiopathic type that arises de novo within the bone [2]. This might be caused by intramedullary metaplasia followed by mucoid degeneration with intraosseous cyst formation [1]. Altered mechanical stress may also lead to intramedullary vascular disturbance and aseptic necrosis. Subsequently, the revitalization of these necrotic areas produces fibroblastic proliferation and mucoid degeneration. The second type of intraosseous ganglion is assumed to occur when there is penetration of extraosseous into the underlying bone [2].

These patients may present with persistent pain that worsens with the use of the affected region. Physical examination may reveal swelling with tenderness; however there are often no abnormal findings [10]. The aetiology of pain is unknown but may be due to the pressure of an expanding intraosseous lesion or the fracture at the periphery of the lesion [1, 3, 9].

On radiographs, the intraosseous ganglia appear as well defined, lytic, oval, or round lesions located in the juxta-articular (subchondral) region with or without cortical expansion and soft-tissue extension. Most of the lesions are small, at 1-2 cm [2, 11]. The presence of communication between the cyst and the joint space has been reported. Using conventional tomography, Menges et al. identified a communication between the lesion and the joint in 75% of cases [12]. However, Schajowicz et al. failed to demonstrate communication on radiographs in any of their 88 cases. The authors suggested the possibility of subsequent obliteration of the connecting channel [2]. We were unable to demonstrate a communication between the lesion and the intra-articular space either intraoperatively or by radiographic studies.

Grossly, the intact cyst is usually seen as smooth or slightly lobulated and bulging, with a white, fibrous outer surface. The external surface might appear gray-blue if the wall is thinned [5]. The microscopic examination of the intraosseous ganglion is identical to its soft-tissue counterpart, revealing a cyst wall of fibrous tissue and fibrocytes with areas of collagenous material [5, 13, 14].

The differential diagnosis of intraosseous ganglion includes unicameral bone cyst, chondromyxoid fibroma, Brodie's abscess, giant cell tumour, fibrous dysplasia, and pigmented villonodular synovitis [10, 15]. In this case the patient underwent surgery with percutaneous drilling for a possible Brodie abscess and had a recurrence of the lesion possibly due to the fact that the cavity was only decompressed and not excised. At the patient's initial presentation the possibility of intraosseous ganglion was not considered in the differential diagnosis of the lesion and an MRI scan to aid in the diagnoses was not available. Microbiology specimens were taken for cultures and sensitivity but histopathological samples to confirm the diagnosis were not.

Generally, the ideal treatment of symptomatic intraosseous ganglion is surgical excision by curettage followed by bone grafting to prevent recurrence and the risk of collapsing fracture [3, 5].

Our patient had recurrence after the initial surgical management. The current literature reveals that recurrence after surgical curettage is rare (recurrence rate 6.1%). However, recurrence is not always related to inadequate excision or curettage. It can be caused by the occurrence of connective tissue metaplasia either at the operative site or in the tissue immediately adjacent to it [16].

## 4. Conclusion

An Intraosseous ganglion is an uncommon lesion. Nevertheless, it is a possible cause of ankle pain and should be included in the differential diagnosis of benign osteolytic lesions in the ankle joint. Imaging in the form of an MRI is essential to aid in diagnosis and operative planning, accompanied with histopathology to confirm the diagnosis. Surgical curettage followed by bone grafting is the treatment of choice.
